# Diseases Admitted under the Care of the Physicians of the Westminster Hospital, from November 26 to December 26, 1801

**Published:** 1802-01

**Authors:** 


*Diseases admitted under the Care of the Physicians of the Westminſter Hoſpital, from November* 26 *to December* 26, 1801.

| | |
|---|---|
| Continued Fever - - - - | 15 |
| Intermittent - - - - - - | 1 |
| Pleuritis - - - - - - - - | 2 |
| Eryſipelas - - - - - - | 1 |
| Cynanche - - - - - - | 3 |
| Anaſarca - - - - - - - - | 6 |
| Anorexia - - - - - - - | 2 |

| | | | |
|---|---|---|---|
| Afcites | 1 | Icterus | 2 |
| Afthenia | 3 | Impetigo | 5 |
| Afthma | 2 | Lepra Græc | 1 |
| Catarrh | 7 | Lumbago | 2 |
| Chorea | 1 | Menorrhagia | 1 |
| Colic | 1 | Palpitatio | 1 |
| Diarrhœa | 14 | Paralyfis | 2 |
| Dyfenteria | 3 | Pertuffis | 2 |
| Dyfpepfia | 11 | Pleurodyne | 5 |
| Dyfpnœa | 3 | Procidentia | 2 |
| Dyfuria | 1 | Prurigo | 1 |
| Enterodynia | 3 | Rheumatifm | 10 |
| Epilepfia | 1 | Scabies | 1 |
| Erythema | 2 | Sciatica | 2 |
| Gaftrodynia | 1 | Struma | 1 |
| Hæmatemefis | 1 | Tinea | 2 |
| Hæmoptoe | 1 | Tremor | 1 |
| Hæmorrhois | 2 | Tuffis | 17 |
| Hemicrania | 1 | Tympanites | 1 |
| Herpes | 2 | Vertigo | 3 |
| Hydrocephalus | 1 | Vomitio | 3 |
| Hyfteria | 5 | Urticaria | 1 |

The great decreafe in the number of Continued Fevers fince the laft account, is the regular and ufual effect of change of feafon in London. During the months Auguft, September, October, and November, our only fevers are Synochus Biliofa. Typhus is almoft unknown at thofe times, becaufe the caufes of it feldom occur. But as foon as the cold weather of winter fets in, and the poor begin to fhut themfelves up in their unventilated garrets and cellars, then Typhus appears, and continues through the fpring. This fpecies of fever is principally diftinguifhed here by the following marks. It fupervenes by imperceptible degrees; when formed, the general debility, defponding countenance, feeble pulfe, tendency to delirium, and dry, hard, brown or black tongue, which trembles on being thruft out, ftrike the attention of the practitioner. It appears always to arife, in the firft inftance, from the miafma generated by the living human body, and afterwards to be propagated like other contagious difeafes.

The Synochus biliofa, on the contrary, commences with chillnefs or fhivering, is not contagious, and the tongue is never dry and hard, as if baked, but moift, and covered with a buff-coloured furr. We are fenfible that thefe prominent marks admit of exceptions; but we deem them fufficient to convey our meaning, and hope the example of Dr. Girdleftone will be followed by our other Correfpondents. [*See Address to Correfpondents at the end of this Number.*]

